# Controversies in the management of cysticercosis.

**DOI:** 10.3201/eid0303.970324

**Published:** 1997

**Authors:** C Evans, H H Garcia, R H Gilman, J S Friedland

**Affiliations:** University of Cambridge Clinical School, Cambridge, United Kingdom.


### Controversies in the Management of Cysticercosis

Cysticercosis, an infection caused by larvae of the pork tapeworm *Taenia solium* in human tissues, is a common cause of neurologic disease in most non-Muslim developing countries, where it accounts for more than one-third of adult-onset epilepsy cases (1). Cysticercosis is increasingly diagnosed in patients in industrialized nations; persons who have never left the United States as well as visitors to disease-endemic regions are at risk. Traditionally considered an exotic disease, this infection now accounts for up to 2% of neurologic/neurosurgical admissions in southern California (2) and more than 1,000 cases per year in the United States (3). Further away from disease-endemic regions, an outbreak of cysticercosis among orthodox Jews living in New York City was reported after food was contaminated with *T. solium* eggs by immigrant cooks infected with the pork tapeworm (4); these carriers may have been completely unaware of their infections. Neurocysticercosis has been reported in AIDS patients, but immuno-suppression does not appear to increase the incidence of this infection. Once cysticercosis is diagnosed, treatment may be necessary, but optimal therapy and particularly the role of cestocidal drugs is controversial. In this commentary, we discuss current options in the treatment of established cysticercosis.

The clinical and pathologic features of neurocysticercosis vary, depending on the inflam-matory response around cysticerci, their number, size, and location. The presence of viable, living cysticerci in the central nervous system usually does not cause symptoms (5). In contrast, inflammation around degenerating cysticerci may have severe consequences, including focal encephalitis, edema, and vasculitis. The most frequent symptom is epilepsy. However, neurocysticercosis can cause a wide variety of clinical syndromes—from chronic meningitis and cranial nerve palsies to spinal infarction and symptoms due to either a mass effect or, particularly in racemose disease, raised intracranial pressure. Such variable clinical features necessitate further investigations to make a diagnosis before treatment.

The diagnosis may be made by excision biopsy of subcutaneous cysticerci, which are found in 4% to 25% of patients with neurocysticercosis (the percentages are higher in Asia than in Latin America). However, radiologic and serologic tests are usually required for diagnosis unless biopsy of a central nervous system lesion is possible. Computed tomography visualizes living cysticerci as hypodense lesions not enhanced with intravenous contrast; a small, hyperdense scolex may be observed within a living cyst (6). Degenerating cysticerci which are more often symptomatic are isodense or hyperdense, and edematous inflammation around them usually causes ring or nodular enhancement by intravenous contrast (5). Magnetic resonance imaging provides detailed images of living and degenerating cysticerci, as seen in a heavily infected patient (Figure), but may not detect calcified, destroyed cysticerci (3). An immunoblot diagnostic test on serum has been shown to have greater than 98% sensitivity and specificity (7). However, in patients with single ring-enhancing lesions, sensitivity falls to 60% to 80%. Sensitivity is also reduced if cerebrospinal fluid rather than serum immunoblot is used.

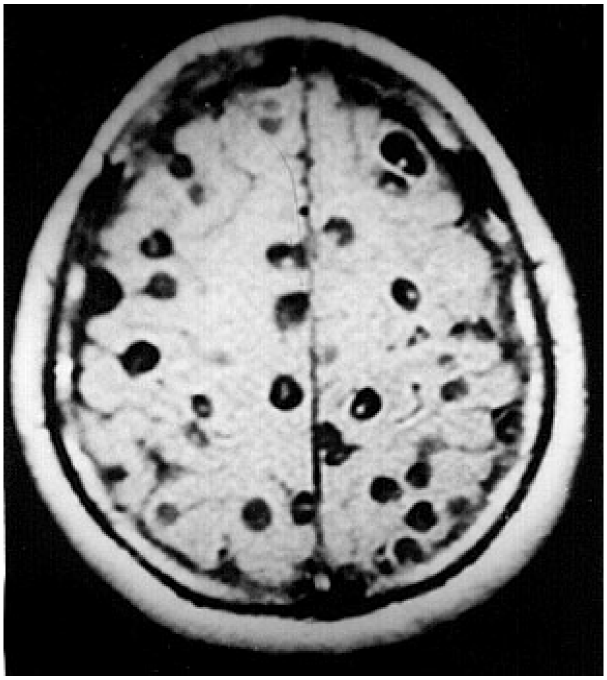

Figure. Magnetic resonance image of a patient with neurocysticercosis demonstrating multiple cysticerci within the brain.

The treatment of established neurocysticercosis is controversial and probably depends on the associated inflammatory reaction as well as clinical and pathologic features. Symptomatic therapy with conventional anticonvulsant drugs is indicated to control epilepsy. Symptoms often result from self-limiting inflammation around a degenerating cysticercus (3,5). Raised intracranial pressure caused by this local reaction usually responds to oral corticosteroids. Steroids have been given chronically in occasional cases of persistent intracranial inflammation. Surgery also has a role: a ventriculoperitoneal shunt relieves obstructive hydrocephalus, although shunt blockage is common when the cerebrospinal fluid protein is elevated. Because inflammation associated with medical therapy may threaten vision, surgery has been used to excise intraocular cysticerci. Asymptomatic subcutaneous or intramuscular cysticerci do not require treatment.

Cestocidal therapy with praziquantel (50 mg/kg/day tid orally for 14 days) or albendazole (15 mg/kg orally tid/bid for 8 to 15 days) accelerates radiologic disappearance of viable intracranial cysticerci. Albendazole may have slightly greater efficacy and is generally less expensive than praziquantel. Cestocidal treatment combined with symptomatic care is associated with a good clinical outcome (8,9). However, these nonrandomized trials were not optimally controlled, and a similarly benign clinical course has been described after symptomatic treatment alone in both adults (5) and children (10). Furthermore, randomized placebo controlled trials with selected patients have shown no clinical (11) or radiologic (12,13) benefit from the addition of cestocidal therapy to symptomatic care. A problem with cestocidal therapy is that it causes influx of inflammatory cells around cysticerci, which is often associated with transient clinical deterioration (8). Rarely, this may be fatal in heavy infections, despite administration of corticosteroids, a common practice to minimize adverse effects (6). Although coadministering corticosteroids reduces blood levels of praziquantel and increases those of albendazole, these effects do not appear to be relevant clinically. Therefore, the immunologic basis has yet to be determined for the inflammation around cysts when they die or are killed by cestocidal treatment (14).

Although recommendations cannot yet be definitive, available evidence suggests that viable, intact cysticerci that cause epilepsy or other symptoms can be treated with cestocidal therapy, especially if they are causing mass effect. If cestocidal treatment is instituted, there is no reason to avoid the use of steroids. These should always be administered before cestocidal therapy is given to patients with multiple viable intracranial cysticerci because the sudden and simultaneous death of these parasites would otherwise cause inflammation, which can be fatal. In contrast, when untreated patients have neurologic symptoms and radiologic evidence of inflammation around a degenerating cysticercus, the parasite has probably already died, and cestocidal therapy is unlikely to be of benefit. In such cases, an expectant policy is reasonable: symptomatic therapy alone for 6 to 12 weeks, unless the patient's condition worsens. A repeat computed tomography scan then usually shows reduction in size or disappearance of a degenerating cysticercus (12,13,15). If improvement has not occurred, then empirical cestocidal chemotherapy may be considered, and possible alternative diagnoses such as tuberculosis should be entertained. Intracranial calcifications and lesions that show ring enhancement on neuroimaging are not living parasites and probably do not warrant cestocidal therapy.

This approach to cestocidal therapy is controversial, and the results of at least one ongoing, double-blind, randomized placebo-controlled trial are keenly awaited. Even when the value of cestocidal therapy is firmly established or refuted, new antiinflammatory treatments will require therapeutic approaches to be reevaluated. A greater understanding of the pathogenesis of this condition is a prerequisite to developing effective therapy to control inflammation around degenerating cysticerci.

### Acknowledgments

The ongoing collaboration between the authors is funded in part by the Wellcome Trust of the United Kingdom, STD4 project PL 950004 from the European Commission, and grant number 1-U01 A135894-01 from the National Institutes of Health, USA.

**Carlton Evans,* Hector H. Garcia,†‡ Robert H. Gilman,‡§ and Jon S. Friedland¶[1]**
*University of Cambridge Clinical School, Cambridge, United Kingdom; †Instituto de Ciencias Neurologicas, Lima, Peru; ‡Universidad Peruana Cayetano Heredia,Lima, Peru; §Johns Hopkins University School of Hygiene and Public Health, Baltimore, USA; and ¶Royal Postgraduate Medical School, Hammersmith Hospital, London, United Kingdom
[1]Corresponding author

[1]Corresponding author

### References

1. Garcia HH, Gilman R, Martinez M, Tsang VCW, Pilcher JB, Herrera G et al. Cysticercosis as a major cause of epilepsy in Peru. Lancet 1993;341:197-200.
2. McCormick GF. Cysticercosis: review of 230 patients. Bulletin of Clinical Neurosciences 1985; 50:76-101.
3. Shandera WX, White AC, Chen JC, Diaz P, Armstrong R. Neurocysticercosis in Houston, Texas. Medicine 1994; 73:37-52.
4. Schantz PM, Moore AC, Munoz JL, Hartman BJ, Schaefer JA, Aron AM, et al. Neurocysticercosis in an orthodox Jewish community in New York City. N Engl J Med 1992; 327:692-5.
5. Kramer LD, Locke GE, Byrd SE, Daryabagi J. Cerebral cysticercosis: documentation of natural history with CT. Radiology 1989; 171:459-62.
6. Wadia N, Desai S, Bhatt M. Disseminated cysticercosis: new observations, including CT scan findings and experience with treatment by praziquantel. Brain 1988; 111:597-614.
7. Tsang VCW, Garcia HH. Immunoblot diagnostic test (EITB) for *Taenia solium* cysticercosis and its contribution to the definition of this under-recognized but serious public health problem. In: Garcia HH, Martinez SM (editors). Teniasis/ Cisticercosis por *T. solium*. Lima: Ed. Universo; 1996. p. 259-69.
8. Sotelo J, Escobedo F, Rodriguez J, Torres B, Rubio F. Therapy of parenchymal brain cysticercosis with praziquantel. N Eng J Med 1984; 310:1001-7.
9. Vasquez V, Sotelo J. The course of seizures after treatment for cerebral cysticercosis. N Engl J Med 1992; 327:696-702.
10. Mitchell WG, Crawford TO. Intraparenchymal cerebral cysticercosis in children: diagnosis and treatment. Pediatrics 1988; 82:76-82.
11. Carpio A, Santillan F, Leon P, Flores C, Hauser A. Is the course of neurocysticercosis modified by treatment with antihelminthic agents? Arch Intern Med 1995; 155:1982-8.
12. Padma MV, Behari M, Misra NK, Ahuja GK. Albendazole in neurocysticercosis. Neurology 1994; 44:1344-6.
13. Padma MV, Behari M, Misra NK, Ahuja GK. Albendazole in single CT ring lesions in epilepsy. Natl Med J India 1995; 8:255-8.
14. Evans C, Gonzalez AE, Gilman RH, et al. Cysticercosis: immunology and immunotherapy. In: Rose FC (editor). Recent advances in tropical neurology. Amsterdam: Elsevier; 1996. p. 155-64.
15. Singhal BS, Ladiwala U. Neurocysticercosis in India. In: Rose FC (editor). Recent advances in tropical neurology. Amsterdam: Elsevier; 1996. p. 99-109.
